# Dclk1 facilitates intestinal tumor growth via enhancing pluripotency and epithelial mesenchymal transition

**DOI:** 10.18632/oncotarget.2393

**Published:** 2014-09-02

**Authors:** Parthasarathy Chandrakesan, Nathaniel Weygant, Randal May, Dongfeng Qu, Harisha R. Chinthalapally, Sripathi M. Sureban, Naushad Ali, Stan A. Lightfoot, Shahid Umar, Courtney W. Houchen

**Affiliations:** ^1^ Department of Medicine, University of Oklahoma Health Sciences center, Oklahoma City, OK 73104, USA; ^2^ OU Cancer Institute, University of Oklahoma Health Sciences center, Oklahoma City, OK 73104, USA; ^3^ Department of Pathology, University of Oklahoma Health Sciences Center, Oklahoma City, OK 73104, USA; ^4^ Department of Veterans Affairs Medical Center, Oklahoma City, OK 73104, USA; ^5^ Department of Molecular and Integrative Physiology, University of Kansas Medical Center, Kansas City, KS, USA

**Keywords:** Dclk1, self-renewal, pluripotency, EMT, miRNAs, tumorigenesis

## Abstract

Doublecortin-like kinase 1 (Dclk1) is overexpressed in many cancers including colorectal cancer (CRC) and it specifically marks intestinal tumor stem cells. However, the role of Dclk1 in intestinal tumorigenesis in *Apc* mutant conditions is still poorly understood. We demonstrate that Dclk1 expression and Dclk1+ cells are significantly increased in the intestinal epithelium of elderly *Apc^Min/+^* mice compared to young *Apc^Min/+^* mice and wild type mice. Intestinal epithelial cells of *Apc^Min/+^* mice demonstrate increased pluripotency, self-renewing ability, and EMT. Furthermore, miRNAs are dysregulated, expression of onco-miRNAs are significantly increased with decreased tumor suppressor miRNAs. In support of these findings, knockdown of Dclk1 in elderly *Apc^Min/+^* mice attenuates intestinal adenomas and adenocarcinoma by decreasing pluripotency, EMT and onco-miRNAs indicating that Dclk1 overexpression facilitates intestinal tumorigenesis. Knocking down Dclk1 weakens Dclk1-dependent intestinal processes for tumorigenesis. This study demonstrates that Dclk1 is critically involved in facilitating intestinal tumorigenesis by enhancing pluripotency and EMT factors in Apc mutant intestinal tumors and it also provides a potential therapeutic target for the treatment of colorectal cancer.

## INTRODUCTION

More than 80% of colorectal cancer (CRC) is associated with the *APC* mutation. *APC* is a tumor suppressor gene that is mutated in patients with familial adenomatous polyposis (FAP) and the majority of sporadic colorectal cancers [1, 2]. *Apc* mutation dysregulates the Wnt signaling pathway and triggers the expansion and transformation of the stem cell compartment, resulting in the development of adenomatous polyps [3]. Because of stem cell self-renewal capability, irreversible or unrepaired alterations in the genomes of these cells can be preserved in their amplified progeny [4, 5]. Therefore, *Apc* mutations in intestinal stem cells may transform these cells and initiate expansion leading to cancer development. Like humans with germline mutations in *APC*, *Apc^Min/+^* mice have a heterozygous mutation in the *Apc* gene, predisposing the mice to intestinal and colon tumor development. These mice start developing intestinal polyps by ~4 weeks of age, with progression to dysplasia at 18–21 weeks of age, adenocarcinoma is also evident at ~26–34 weeks of age [6–9]. Younger *Apc^Min/+^* mice (8–12 weeks of age) are good models to study the pathogenesis of FAP, while elderly *Apc*^Min/+^ mice (26–34 weeks of age) develop intestinal low and high-grade dysplasia and adenocarcinoma and are relevant models for studying tumor progression, as well as developing good therapeutic strategies [7, 8]. Elderly *Apc*^Min/+^ mice are a particularly clinically relevant disease model because a large percentage of patients diagnosed with advanced colon cancer have an unresectable or widespread disease [10].

Doublecortin-like kinase 1 (Dclk1) is a member of the protein kinase super family and the doublecortin family. Dclk1 is overexpressed in many cancers, including colon, pancreas, liver and esophagus [11–14]. Recent studies show that Dclk1 specifically marks tumor stem cells (TSCs) that self-renew and generate tumor progeny in *Apc^Min/+^* mice [15]. It has been also shown that the development and progression of pancreatic cancer depend upon Dclk1+ TSCs [16]. Previous work from us and others supported that DCLK1 expression in cancer is critical for cancer growth, EMT, and metastasis [11, 12, 16–19]. Studies indicate that gain of stem cell-like properties are essential features of epithelial-mesenchymal transition (EMT), a process that plays a key role in cancer progression and metastasis [20]. The functional interdependence between EMT-associated transcription factors and enhanced self-renewal ability highlights the common mechanism involved in tumorigenesis. However, the potential roles of Dclk1 in *Apc* mutant conditions for facilitating intestinal tumorigenesis are yet to be well known.

## RESULTS

### Increased expression of Dclk1 and Dclk1+ cells in the intestine of *Apc^Min/+^* mice is associated with adenoma and adenocarcinoma

**Figure 1:**
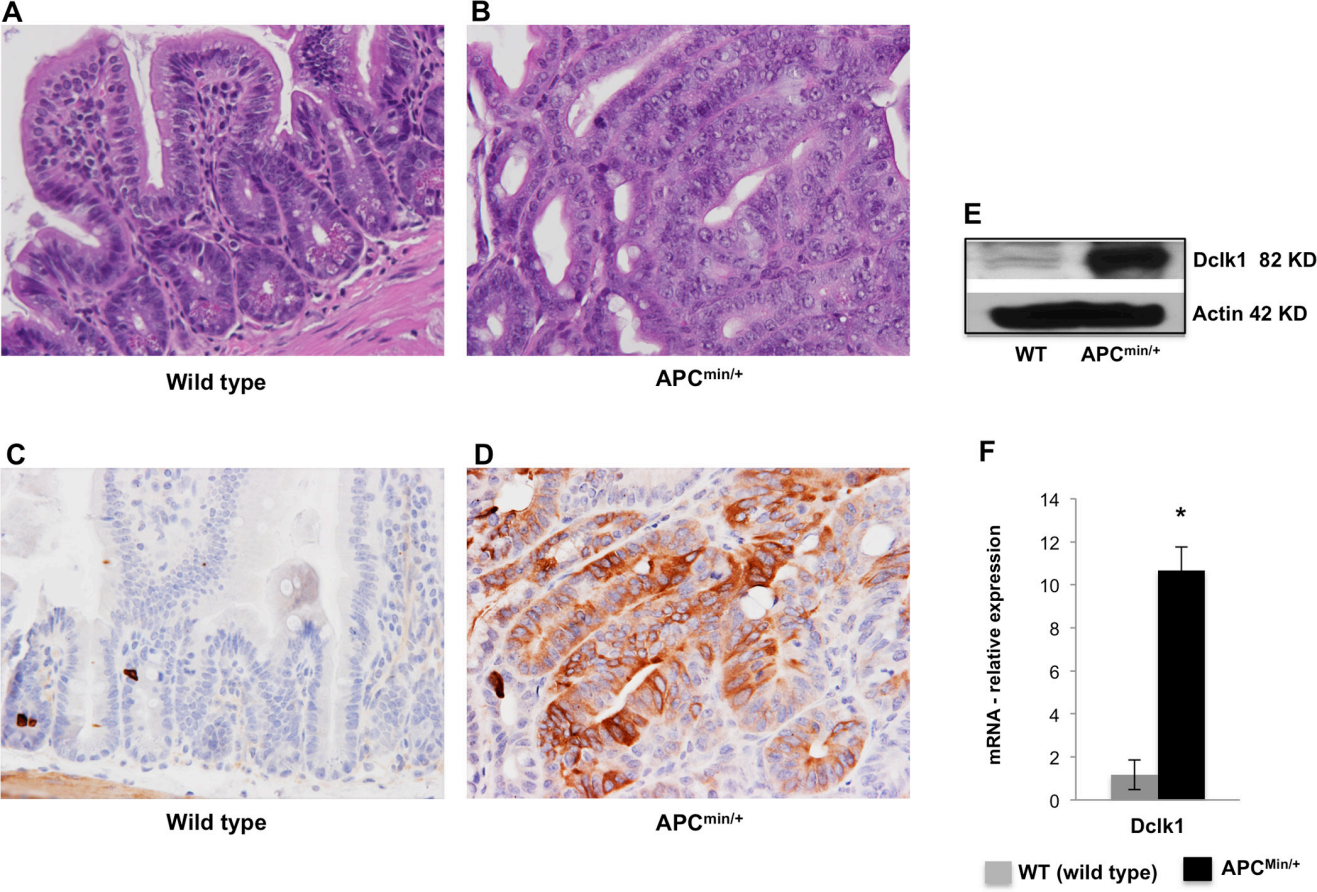
Dclk1 is overexpressed in the intestinal adenomas and adenocarcinomas H&E staining of the SI shows normal crypt architecture in WT and aberrant crypt architecture and dysplasia in 30 weeks old *Apc^Min/+^* mice. H&E in the small intestine of WT **(A)** and *Apc^Min/+^* mice **(B)**, (n=5; magnification: 400X). IHC for Dclk1 in the small intestine of WT **(C)** and *Apc^Min/+^* mice **(D)**, (n=5; magnification: 400X), resulted in a massive increase in the Dclk1+ cell population in *Apc^Min/+^* mice. The increase happened in particular regions identified as high grade dysplasia and adenocarcinoma where more cells were positive for Dclk1. There were differences in the number of Dclk1+ cells in staining corroborates with protein and mRNA levels of Dclk1 in isolated IECs of WT and *Apc^Min/+^* mice (E&F). All quantitative data are expressed as means ± SD of minimum three independent experiments. P values <0.05 were considered statistically significant.

To better dissect the role of Dclk1 in intestinal tumorigenesis, we analyzed the intestinal crypt architecture and expression of Dclk1, pluripotency, and EMT associated factors between 12 week old and 30 week old *Apc^Min/+^* mice. Moreover, to determine the significance of the Dclk1+ cell in intestinal tumorigenesis, we assessed whether Dclk1+ cells were expanded in *Apc^Min/+^* mice at 12 and 30 weeks of age. H&E staining shows that the intestinal epithelium having hyperplastic crypts, polyps, and no sign of dysplasia and/or adenocarcinoma in 12 week old *Apc^Min/+^* mice compared to 30 week old *Apc^Min/+^* mice, which had intramucosal adenocarcinoma with low and high-grade dysplasia. The crypt architecture is distorted with no identifiable crypt structures in the places where we identified adenocarcinoma and high-grade dysplasia (Figure 1B and Supplementary Figure 1A, B, C, D). As expected, the intestinal crypt architecture of wild-type (WT) mice appeared normal (Figure1A). IHC staining revealed 5–10% of Dclk1+ cells in the intestine of 12 week old *Apc^Min/+^* mice (Supplementary Figure 2), whereas, large populations of Dclk1+ cells (25–30%) were found in the intestines of the 30 week old *Apc^Min/+^* mice (Figure 1D and Supplementary Figure 2). These observations suggest that Dclk1+ cells started expanding before 12 weeks of age, whereas greater populations of Dclk1+ cells at 30 week of age may represent clonal Dclk1+ neoplastic cells that have expanded during the process of tumorigenesis. In confirmation of our previous studies [11], immunohistochemical (IHC) staining of Dclk1 in the WT intestines revealed approximately 1–3% Dclk1+ cells (Figure 1C). To verify that the Dclk1 upregulation in tumors of *Apc* mutation with activated wnt, we did IHC for β-catenin and found that β-catenin was localized in the intestinal regions identified as high-grade dysplasia and adenocarcinoma, and most of them strongly stained in the nucleus (Supplementary Figure 3A). We also found the protein expression of β-catenin and its downstream molecule TCF4 increased in the IECs of *Apc^Min/+^* mice compared to WT control (Supplementary Figure 3B). These findings suggest that both β-catenin and Dclk1 in tumor lesions of *Apc^Min/+^* mice showed a progressive increase during tumorigenesis. Furthermore, we found that Dclk1 expression was found to be ~10 fold higher and the pluripotency and EMT associated factors were massively increased in the IECs of 30 week old *Apc^Min/+^* mice (Figure 1E, 1F and Supplementary Figure 4 and 5) compared to 12 week old *Apc^Min/+^* mice (Supplementary Figure 4 and 5). These observations suggest that *Apc^Min/+^* mice at 12 weeks of age provide a good platform to understand early tumorigenesis, whereas *Apc^Min/+^* mice at 30 weeks of age provide a compelling platform to understand the molecular events associated with advanced intestinal tumorigenesis. In this study, elderly *Apc^Min/+^* mice at 30 weeks of age were used along with age and sex matched WT littermates to assess the molecular events associated with advanced intestinal tumorigenesis, and to determine the efficiency of targeted therapy in advanced diseases.

### Dclk1 upregulation in intestinal epithelial cells is associated with increased pluripotency and EMT

**Figure 2:**
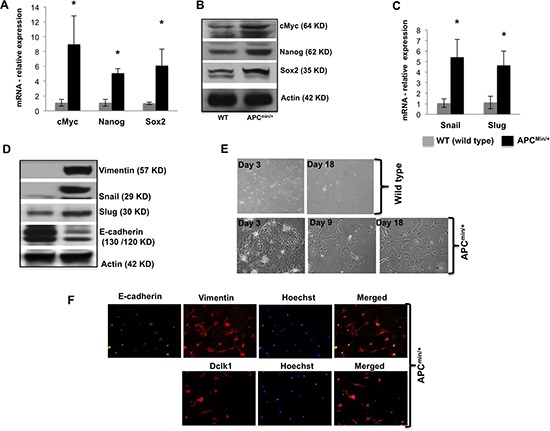
Dclk1 upregulation is associated with enhanced pluripotency and EMT Pluripotency factors including cMyc, Nanog, and Sox2 were all increased in mRNA and protein expression level in the isolated IECs of *Apc^Min/+^* mice compared to WT mice **(A&B)**. Protein and mRNA expression of EMT-associated factors, Snail, Slug, and protein expression levels of Vimentin and E-cadherin in the IECs of WT and *Apc^Min/+^* mice **(C&D)**. Primary cell culture and monolayer formation of isolated IECs of WT and *Apc^Min/+^* mice in vitro for EMT analysis **(E)**. Immunofluorescence staining of Vimentin, E-cadherin, and Dclk1 **(F)**, show the onset of EMT in IECs of *Apc^Min/+^* mice. All quantitative data are expressed as means ± SD of minimum three independent experiments. P values <0.05 were considered statistically significant.

To determine the enrichment of pluripotency associated with increased Dclk1 expression during intestinal tumorigenesis, we analyzed the expression of pluripotency factors, and found a massive increase in mRNA and protein levels of the Myc, Nanog and Sox2 (Figure 2A and 2B) in the 30 week old *Apc^Min/+^* mice IECs compared to age and sex matched WT control mice, confirming greater self-renewal ability of IECs during tumorigenesis. We also performed IHC for Nanog on intestinal tissue sections of 30 weeks old *Apc^Min/+^* mice and found that the nanog staining was increased in the intestinal regions identified as high-grade dysplasia and adenocarcinoma (Supplementary Figure 3D). To examine the onset of EMT during tumorigenesis, we examined IEC monolayer-forming ability. Only the IECs isolated from *Apc^Min/+^* mice formed monolayers and revealed significant transdifferentiation into cells with mesenchymal characteristics (Figure 2E). These cells stained positive for Vimentin and E-cadherin were decreased and lost from the cell surfaces, while moderately transdifferentiated or transdifferentiating cells were stained positive for both vimentin and E-cadherin (Figure 2F). Moreover, most of the cells were also positive for Dclk1 (Figure 2F), suggesting EMT may be driven in stem-like cells or by stem cells themselves. To better dissect at the molecular level associated with the onset of EMT, we evaluated the expression of EMT-associated transcription factors and found that Slug, Snail and Vimentin were all higher and E-cadherin was lower in the IECs of *Apc^Min/+^* mice compared to WT (Figure 2C and 2D). Furthermore, the staining of snail in the intestine of *Apc^Min/+^* mice was greater in the intestinal regions identified as high-grade dysplasia and adenocarcinoma (Supplementary Figure 3C). Therefore our data suggest that intestinal cellular transdifferentiation is increased with Dclk1 upregulation during tumorigenesis.

### Dclk1+ cells from the intestine of *Apc^Min/+^* mice acquire increased self-renewal ability

**Figure 3:**
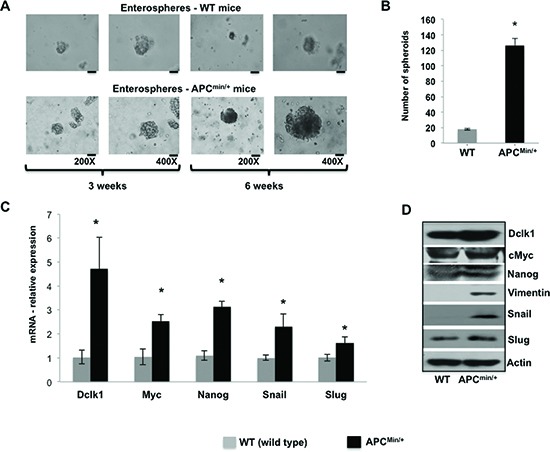
Expansion of Dclk1+ cells and its association to enhanced tumor stemness Enterosphere formation of isolated Dclk1+ cells from the small intestine of WT and *Apc^Min/+^* mice at 200 and 400X magnification **(A)**, bar graph represents the quantification of number of enterospheres formed from Dclk1+ cells isolated from WT and *Apc^Min/+^* mice **(B)**. Characterizing enterospheres formed from Dclk1+ cells for cellular transformation to support tumorigenesis: mRNA and protein expression of Dclk1, EMT, (Slug, Snail, and Vimentin) and pluripotency (Myc andNanog) factors in the enterospheres formed from Dclk1+ cells of WT and *Apc^Min/+^* mice **(C&D)**. All quantitative data are expressed as means ± SD. P values <0.05 were considered statistically significant.

We found upregulated Dclk1 expression and a massive increase in Dclk1+ cells in *Apc^Min/+^* mice intestinal epithelium (Figure 1D, 1E and Supplementary Figure 2). FACS analysis for percent Dclk1+ cells and Dclk1 enrichment in Dclk1+ cells further confirms and validates the enhanced Dclk1+ cells during tumorigenesis in the *Apc^Min/+^* mice (Supplementary Figure 6). Dclk1+ cells isolated from the small intestine of the *Apc^Min/+^* mice formed an average of 150 enterospheres per high power field (HPF) versus 14 per HPF in WT (Figure 3A and 3B). To answer whether enterospheres formed from the Dclk1+ cells of *Apc^Min/+^* mice that were enriched with pluripotency and EMT to support self-renewal ability, we collected enterospheres and analyzed them for EMT and pluripotency factors. We found significantly higher levels of Dclk1 in the enterospheres of *Apc^Min/+^* mice compared to WT (Figure 3C and 3D). More excitingly the expression levels of the EMT-associated factors Snail, Slug, Vimentin, and the pluripotency factors Myc and nanog were significantly increased in the enterospheres of *Apc^Min/+^* mice compared to WT (Figure 3C and 3D). This data suggests that this cellular transformation may endow Dclk1+ cells with greater self-renewal ability and initiate their tumor stem cell function.

### Dysregulation of miRNAs Mediates Cellular Transdifferentiation Towards EMT and Neoplasia

**Figure 4:**
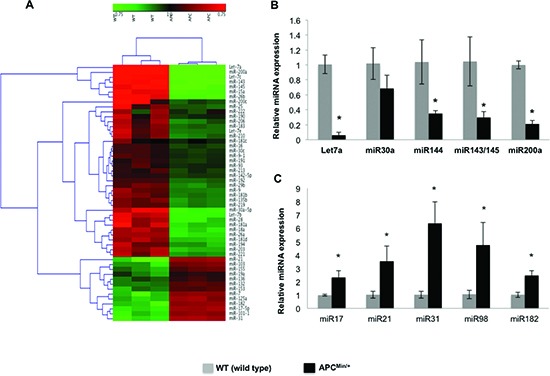
Dysregulation of miRNAs in the intestinal epithelial cells of *Apc^min/+^* mice Profiling of miRNAs on Signosis Cancer MicroRNA Array were performed and analyzed using Genesis software package version 1.7.6. for hierarchical clustering **(A)**, miRNA quantification by miRNA specific RT-PCR, tumor suppressor miRNAs **(B)** and tumor promoter miRNAs **(C)**. All quantitative data are expressed as means ± SD of minimum three independent experiments. P values <0.05 were considered statistically significant.

MicroRNAs (miRNAs) are potentially important for stem cell pluripotency and differentiation, and for complex cellular expression networks in development and disorders [21, 22]. Using Mouse miRNA Arrays (Signosis), miRNAs were identified that were differentially expressed between the IECs of *Apc^Min/+^* and WT control mice (Figure 4A). Hierarchichal clustering of the miRNA data revealed significant upregulation of tumor promoter miRNAs (miR-17, miR-21, miR-31, miR-98 and miR-182) and significant downregulation of tumor suppressor miRNAs (Let7a, miR-143, miR-144, miR145, miR-30a and miR-200a) in the IECs of *Apc^Min/+^* mice (Figure 4A). Those that were most significantly altered as listed above were quantitatively assessed using miRNA specific RT-PCR analyses. Quantitative analysis confirmed their expression signatures, and the listed tumor promoter miRNAs were significantly increased and the tumor suppressors were decreased in the IECs of *Apc^Min/+^* mice compared to WT (Figure 4B, 4C).

### Dclk1 is critically involved in facilitate intestinal tumorigenesis in *Apc^Min/+^* mice

**Figure 5:**
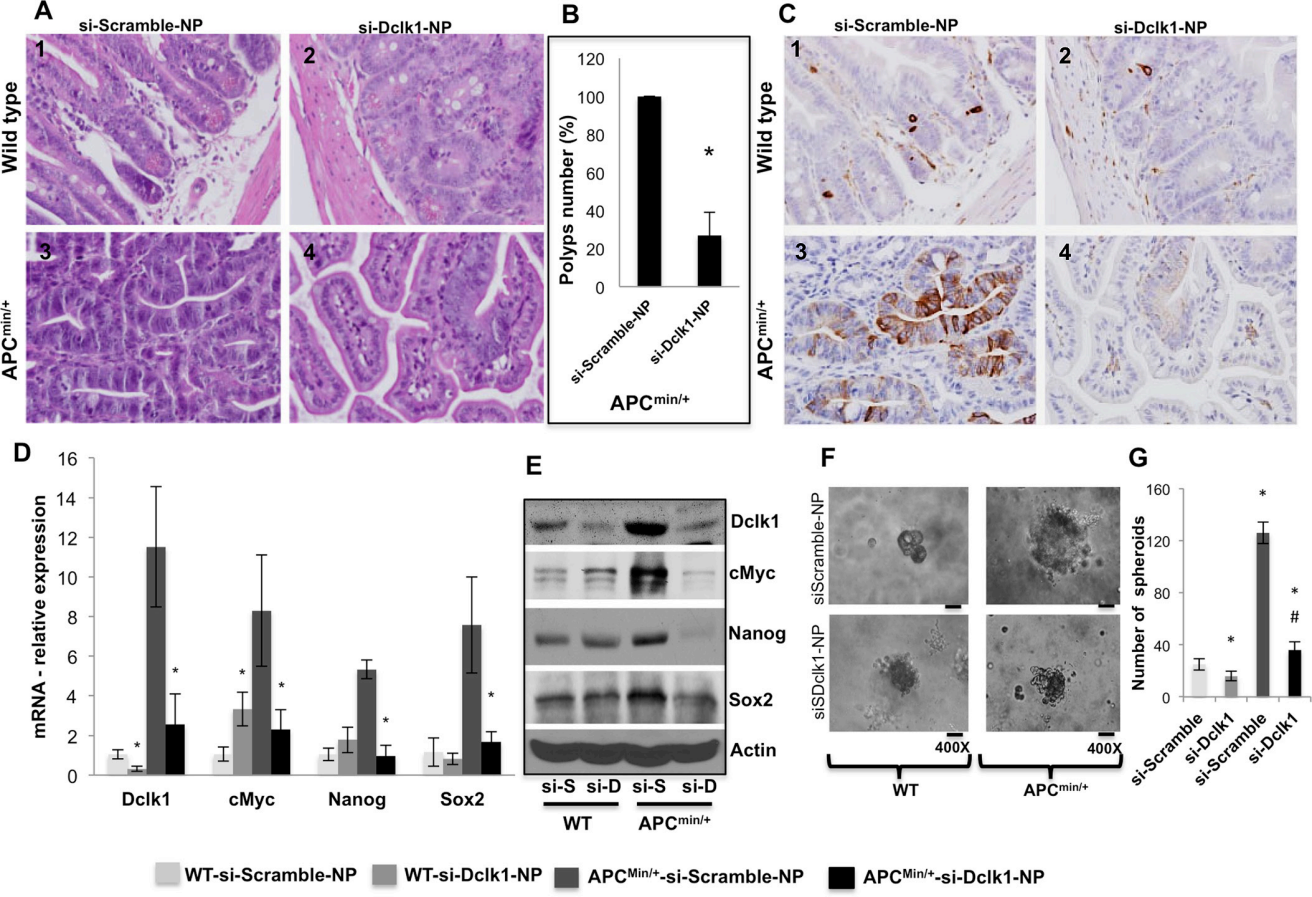
*Dclk1* knockdown decreases polyps and dysplasia/adenocarcinoma and tumor stemness WT and *Apc^Min/+^* mice were treated with siScr-NPs and siDclk1-NPs. H&E staining of small intestines from WT and *Apc^Min/+^* mice (n=5; magnification: 400X (1–4) **(A)**, bar graph represents the quantification in percentage of number of polyps reduced after siDclk1-NPs treatment to *Apc^Min/+^* mice **(B)**. IHC for Dclk1 in the small intestine of WT and *Apc^Min/+^* mice (n=5; magnification: 400X (1–4)) **(C)**. Pluripotency factors were all decreased at mRNA and protein expression level in the isolated IECs of *Apc^Min/+^*mice treated with siDclk1-NPs compared to siScr-NPs (D&E). Clonogenic assay shows enterosphere formation of isolated Dclk1+ cells from the small intestine of WT and *Apc^Min/+^* mice treated with siDclk1-NPs and siScr-NPs (200 and 400X magnification). *Dclk1* knockdown in *Apc^Min/+^* mice reduced the self-renewal ability of Dclk1+ cells **(F)**, bar graph represents the quantification of number of enterospheres formed from Dclk1+ cells isolated from WT and *Apc^Min/+^* mice treated with siDclk1-NPs and siScr-NPs **(G)**. All quantitative data are expressed as means ± SD of three independent experiments. P values <0.05 were considered statistically significant.

**Figure 6:**
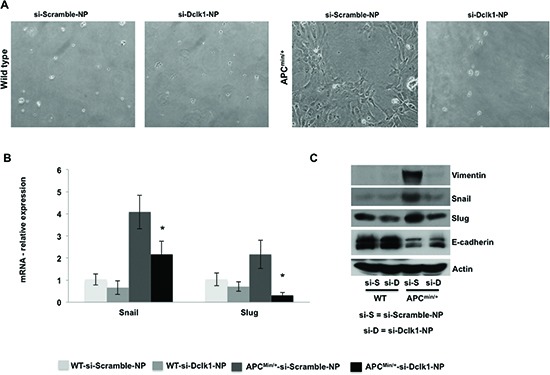
knockdown of *Dclk1* decreases the process of EMT in the IECs of *Apc^Min/+^* mice Primary cell culture and monolayer formation of isolated IECs of WT and *Apc^Min/+^* mice after treatment of siDclk1-NPs or siScr-NPs **(A)**. Protein and mRNA expression levels of EMT-associated factors, Snail and Slug, protein expression of Vimentin and E-cadherin in the IECs of WT and *Apc^Min/+^* mice after treatment of siDclk1-NPs or siScr-NPs **(B&C)**. All quantitative data are expressed as means ± SD of minimum three independent experiments. P values <0.05 were considered statistically significant.

**Figure 7:**
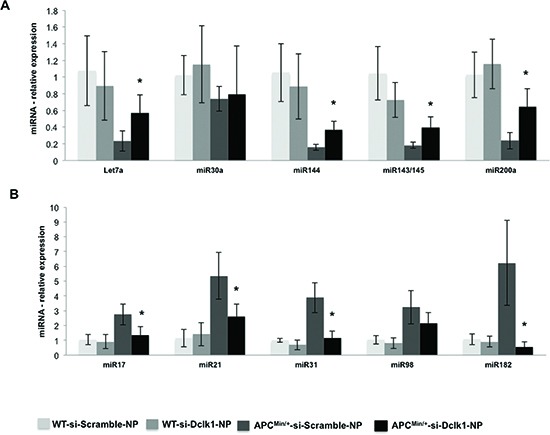
*Dclk1* knockdown decreases the dysregulation in miRNAs in the IECs of *Apc^min/+^* mice Knocking down Dclk1 reduced the dysregulation in the miRNAs expression in the isolated IECs of *Apc^Min/+^* mice compared to siScr-NPs treatment (A&B). Quantitative analysis of the tumor suppressor miRNAs **(A)** and tumor promoter miRNAs **(B)** in the IECs of WT and *Apc^Min/+^* mice treated with siScr-NPs and siDclk1-NPs. *Dclk1* knockdown reduced dysregulation in the miRNAs expression and maintained the expression at physiological or at near physiological levels. *Dclk1* knockdown decreased the onco-miRNAs, and interestingly increased the expression of tumor suppressor miRNAs in the IECs of *Apc^Min/+^* mice compared to siScr-NPs treatment. All quantitative data are expressed as means ± SD of three independent experiments. P values <0.05 were considered statistically significant.

To determine whether Dclk1 is critical for intestinal tumorigenesis, we inhibited *Dclk1* gene expression using siDclk1-NPs, along with si-Scramble-Nanoparticles (siScr-NPs) as the control, in WT and *Apc^Min/+^* mice. Histological studies revealed significantly fewer polyps and decreased dysplasia in the intestine of *Apc^Min/+^* mice treated with siDclk1-NPs compared to siScr-NPs, whereas WT mice did not have any abnormality or change in crypt architecture (Figure 5A and 5B). This data shows that Dclk1 inhibition reduces intestinal tumor formation and growth in *Apc^Min/+^* mice. IHC staining of Dclk1 (Figure 5C) showed a massive decrease in the number of Dclk1+ cells in the small intestine of *Apc^Min/+^* mice treated with siDclk1-NPs compared to siScr-NPs. Significantly lower expression levels of Dclk1, and the pluripotency factors Myc, Sox2, and Nanog were detected in the isolated IECs of siDclk1-NP–treated *Apc^Min/+^* mice (Figure 5D and 5E). Interestingly, the self-renewal ability of Dclk1+ cells and their populations were lowered with siDclk1-NP treatment, as evidence shown by fewer and smaller enterospheres formed from Dclk1+ cells of *Apc^Min/+^* mice and a decreased number of Dclk1+ cells by IHC analysis (Figure 5F, 5G and 5C). IECs from *Apc^Min/+^* mice treated with siScr-NPs formed monolayers (Figure 6A) demonstrating active EMT processes while the IECs of *Apc^Min/+^* mice treated with siDclk1-NPs failed to form monolayers. In addition, Slug, Snail, and Viment in levels were lower and E-cadherin levels were marginally higher in the IECs of *Apc^Min/+^* mice treated with si-Dclk1-NPs (Figure 6B and 6C). Quantitative analysis of tumor suppressor and tumor promoter miRNAs revealed most were at normal or near-normal levels after Dclk1-knockdown (Figure 7A and 7B). Tumor suppressors miRNAs levels increased and tumor promoter miRNAs levels decreased after siDclk1-NP treatment in *Apc^Min/+^* mice. These data suggest that Dclk1 is critically involved in facilitating intestinal tumorigenesis by enhancing pluripotency, EMT associated factors, self-renewal ability, and onco-miRNAs.

## DISCUSSION

*Apc^Min/+^* mice are excellent models to evaluate human FAP and sporadic CRC [1, 2, 8]. We used *Apc^Min/+^* mice at 30 weeks of age, which exhibit high-grade dysplasia and intramucosal adenocarcinoma (Supplementary Figure 1) to improve our understanding of the molecular events associated with advanced intestinal tumorigenesis. Multiple intestinal tumor onset and progression in the elderly *Apc^Min/+^* mice allowed us to investigate how Dclk1 supports intestinal tumorigenesis and to identify novel strategies for cancer prevention and potential treatment. We detected overexpression of Dclk1 in small IECs of *Apc^Min/+^* mice, suggesting that Dclk1 marks intestinal TSCs [17], which expands during intestinal tumorigenesis. These findings agree with our previous studies using human cancer samples of colon, liver, pancreas and esophagus, where Dclk1+ cells were expanded [11–14, 23]. In this study, we provide evidence that loss of *Apc* significantly increases the number of Dclk1+ cells in the small intestine and particularly in dysplastic and adenocarcinoma regions of advanced polyps, supporting the previous hypotheses that (i) stem like cells or stem cells are more abundant in cancerous conditions and (ii) the loss of function of *Apc* increases the expansion of the TSC compartment [3, 24].

Pluripotency is a central, well-defined feature of stem cells and EMT plays a key role in the increase of stem-like cells during tumorigenesis [20, 25]. Greater pluripotency, EMT capacity and higher Dclk1 expression in the IECs of the *Apc^Min/+^* mice in the present study points out a common mechanism of functional interdependence between Dclk1 and pluripotency and EMT factors that may increase stem cell compartment during intestinal tumorigenesis similar to that observed in breast cancer [26]. The expression of Vimentin, loss of E-cadherin, and increased expression of EMT associated transcriptional factors snail and slug, together lead to changes in cellular morphology towards mesenchymal features that further support a role for Dclk1 in the EMT process during intestinal tumorigenesis. Furthermore, more and larger enterospheres formed from the intestinal Dclk1+ cells of *Apc^Min/+^* mice. Molecular characterization of these enterospheres demonstrated enhanced pluripotency and EMT signaling pathways with greater self-renewal ability, which supports the process of cellular transformation into tumor cells and or TSCs. Thus, we hypothesize that the increase in Dclk1 associated with the loss of *Apc* escalate cellular transformation and stem cell compartment to facilitate expansion of dysplasia and adenocarcinoma in tumor-initiated intestinal epithelium.

Dysregulated miRNAs signalings that control EMT, pluripotency and acquired self-renewal capacity for cellular transformation are required for tumorigenesis [21, 27]. Most interesting observation is the dysregulation of miRNAs identified as tumor promoters and suppressors in the IECs of *Apc^Min/+^* mice. However, after the treatment of siDclk1-NP, the dysregulated miRNAs were decreased and or maintained at physiological levels in the *Apc^Min/+^* mice which led us to hypothesize that Dclk1 may regulate the miRNAs biogenesis or signaling to enhance the pluripotency, EMT, and TSCs function to facilitate intestinal tumorigenesis. However, further molecular studies are warranted to demonstrate the link between these requisite molecular alterations, which are necessary for the onset of EMT, and increased pluripotency, to support intestinal tumorigenesis and TSC functions [21, 22, 28].

To demonstrate the functional significance of Dclk1 in intestinal tumorigenesis, we conducted Dclk1 knockdown experiments with siDCLK1-NPs [29] and found decreased dysplasia/adenocarcinoma, and fewer polyps in *Apc^Min/+^* mice. However, the crypt architecture in WT littermates were unaffected. These findings suggest that Dclk1 reduces tumor formation and progression in *Apc^Min/+^* mice without affecting normal epithelial homeostasis. Our data shows that ablation of Dclk1 expression results in the regression of polyps and dysplasia without injury to the normal intestine, suggesting that Dclk1 maybe a potential therapeutic target in intestinal cancer. Recently, the Chiba group found out that specific ablation of Dclk1+ TSCs results in the regression of polyps without injury to the normal intestine [17]. Our data also supports this observation. We found that dysregulated miRNAs signature, had largely reverted to WT physiological levels in the IECs of *Apc^Min/+^* mice after Dclk1 knockdown. Therefore, our data combined with previous findings that Dclk1 knockdown induces tumor growth arrest [12, 29] suggesting that Dclk1 is critical for intestinal neoplasia. Indeed, expression levels of pluripotency factors and self-renewal ability were decreased in the IECs of *Apc^Min/+^* mice following Dclk1 knockdown. Interestingly, knocking down Dclk1 in *Apc^Min/+^* mice resulted in lower expression of factors associated with EMT and in fewer and small erenterospheres. These data suggest that Dclk1 knockdown diminishes dysregulation in miRNAs, EMT, and pluripotency signaling responsible for cellular transformation, enhanced self-renewal ability, and stem cell compartment required for the advancement of intestinal neoplasia.

In conclusion, we have demonstrated that Dclk1 is critically involved in facilitating intestinal tumorigenesis during loss of function of *Apc*. We also demonstrated that Dclk1 supports intestinal tumor growth via enhancing EMT and pluripotency factors. We hypothesize that Dclk1 enhances the EMT and pluripotency factors by regulating the biogenesis of miRNAs. However, additional molecular studies are warranted to demonstrate the link between Dclk1 and miRNAs. The APC and Dclk1 axis is critical for increased stem cell compartment with enhanced self-renewal ability for the advancement of intestinal tumorigenesis. Targeting Dclk1 with siDCLK1-NPs reduces the dysregulation in miRNAs, EMT, and pluripotency associated with cancer risk, suggesting that targeting Dclk1 in patients even with advanced cancer may be a therapeutic option for intestinal and/or other solid tumors.

## MATERIALS AND METHODS

### Animals

All animal experiments were performed with approval and authorization from the Institutional Review Board, and the Institutional Animal Care, and Use Committee at the University of Oklahoma Health Science Center (Oklahoma City, Oklahoma). *Apc*^Min/+^ mice that had a C57BL/6J background were obtained from The Jackson Laboratory and maintained by breeding *Apc*^Min/+^ males with C57BL/6J females. Mice were genotyped to identify carriers of the *Min* allele of *Apc* with a PCR assay. Same sex matched littermates of C57BL/6J *Apc^Min/+^* and *Apc*^+/+^ mice at 12 and 30 week of age were used in the present study. It has been shown that the average life span of *Apc*^Min/+^ mice on C57BL/6J background is ~20 weeks, whereas the mice in our facility have healthier survival rates. This was also observed in several previous studies [6–9]. Elderly *Apc*^Min/+^ mice (i.e., >30 weeks of age) were carefully monitored and sacrificed before becoming moribund.

### Intestinal Epithelial cell (IEC) Isolation and Monolayer Formation

Small intestines were attached to a paddle, immersed in Ca^2+^-free standard Krebs-buffered saline (in mmol/liter: 107 NaCl, 4.5 KCl, 0.2 NaH_2_PO_4_, 1.8 Na_2_HPO_4_, 10 glucose, and 10 EDTA) at 37°C for 15–20 min, and gassed with 5% CO_2_, 95% O_2_. Individual crypt units were then separated by intermittent (30 sec) vibration into ice-cold phosphate buffered saline and were then collected by centrifugation [30–33]. The pellets were washed with phosphate-buffered saline, resuspended in RPMI glutamax medium/0.5 U/ml, dispased at 37°C, and shaken gently for 5 min. The cells were pelleted and resuspended in RPMI glutamax medium supplemented with 5% fetal calf serum, plus penicillin and streptomycin and incubated at 37°C in 5% CO_2_. Monolayer formation was followed for 0–20 days and medium was replaced every 72 hours [32]. Only the IECs isolated from *Apc^Min/+^* mice formed monolayers and exhibited mesenchymal characteristics. These changes were not driven by contamination from the mesenchymal cells during the isolation process, since the purified small intestinal epithelial cells were negative for α-smooth muscle actin expression in WT and *Apc*^Min/+^ mice (Data not shown).

### FACS

Freshly isolated IECs were washed and resuspended in RPMI glutamax medium. To avoid endothelial and stromal contamination, isolated cells were incubated with anti-CD45, anti-CD31, and anti-EpCAM in addition to anti-Dclk1 antibodies conjugated with respective fluorochromes for 30 min. The cells were washed and sorted using Influx-V cell sorter (Cytopeia). CD45-CD31-EpCAM+Dclk1+ cells were then collected and subjected to enteropshere assays.

### Enterosphere formation Assay

Isolated IECs were plated at a density of 1000 cells/well in 48-well plates in RPMI medium containing 0.3% soft agar and 2% fetal calf serum. The cell suspensions were plated in a 48-well plate above a layer of solidified 1% soft agar in plain RPMI medium. The plates were then incubated at 37°C under 5% CO_2._ Then the cells were being monitored for spheroid formation in RPMI glutamax medium plus 1% fetal calf serum with 1X Insulin/Transferrin/Sodium selenite (ITS) and 10,000 Units/ml IFN-gamma at weekly intervals for 5–8 weeks [32].

### MicroRNA Array and Quantitative analysis

Total miRNA was isolated from small intestinal epithelial cells using the miRNeasy mini kit (Qiagen, CA, USA), following the manufacturer's protocol. MicroRNA profiling was performed on the Signosis Cancer MicroRNA Array platform, which contained capture probes for all miRNAs annotated in miRBase (version 15.0; http://www.mirbase.org/). The data generated was then weighted, log base 2 converted, and analyzed. Heat maps were prepared using the Genesis software package, version 1.7.6. For quantitative analysis, total miRNA isolated from intestinal epithelial cells were subjected to reverse transcription with Superscript™ II RNase H - Reverse Transcriptase and random hexanucleotide primers (Invitrogen, Carlsbad, CA). Complementary DNA (cDNA) was subsequently used to perform real-time PCR with SYBR™ chemistry (Molecular Probes, Eugene, OR) using specific primers for selected miRNAs (Supplemental Table 1). The crossing threshold value assessed by using real-time PCR was noted for the transcripts and normalized with *U6* pri-miRNA. The changes in pri-miRNAs were expressed as fold changes relative to the control value ± SD.

### RNA Isolation and Real-time RT-PCR Analysis

Total RNA isolated from small intestinal epithelial cells were subjected to reverse transcription and the complementary DNA (cDNA) was subsequently used to perform real-time PCR with SYBR™ chemistry (Molecular Probes, Eugene, OR) using gene-specific primers (Supplemental Table 2) for specific transcripts. The crossing threshold value assessed by real-time PCR was noted for the transcripts and normalized to internal control.

### Immunoblot Analysis

Standard immunoblot protocols were used. Twenty-five micrograms of the total protein was size separated in an 8%–12% SDS polyacrylamide gel and transferred electrophoretically onto a PVDF membrane with a wet-blot transfer apparatus (Bio-Rad, Hercules, CA). The membrane was then blocked with 5% milk and incubated overnight with a primary antibody (used recommended dilutions by manufacturers) and then with horseradish peroxidase-conjugated secondary antibody (dilution 1:5000). The proteins were detected using ECL Western blotting detection reagents (Amersham-Pharmacia, Piscataway, NJ). Actin (42-kD) was then used as a loading control.

### Small interfering RNAs

Dclk1 siRNA (siDclk1) (Cat. # S234357) sequence targeting the coding region of Dclk1 (accession No. NM_019978) and scrambled siRNAs (siScr) (Cat. # AM4636) did not match any of the mouse genes that were obtained (AmbionInc, Austin, TX).

### Synthesis and characterization of Dclk1 siRNA NPs and treatment

Poly (lactide-*co*-glycolide) acid nanoparticles (PLGA NPs) were synthesized using a double emulsion solvent evaporation technique as described previously [29]. The amount of encapsulated siRNA was quantified using a spectrophotometer (DU-800, Beckman Coulter, Brea, CA). The size, polydispersityindex, and zeta-potential measurements of synthesized siRNA NPs were determined using diffraction light scattering (DLS) and utilizing Zeta PALS (Brookhaven Instruments, Holtsville, NY). Sex and age matched littermates of C57BL/6J *Apc^Min/+^* and *Apc*^+/+^ mice at 30 weeks age, were injected i.p. with 0.25 nmol of siRNA preparation on every third day for a total of 6 doses.

### Immunohistochemistry/immunofluorescence

Standard immunohistochemistry and immunofluorescence protocols were used with specific antibodies.

### Antibodies

We used the following antibodies: Dclk1, Lgr5, Bmi1, Hes1, Tcf4, Snail, Slug, EpCam, CD45, CD31 (all from Abcam, Cambridge, MA), Vimentin, β-catenin (from Santa Cruz Biotechnology), ECadherin, CyclinD1, Nanog, cMyc, Oct4, Sox2, β-actin (all from Cell Signaling, Danvers, MA), Anti-rabbit IgG, Anti-mouse IgG, Anti-goat IgG (Jackson Immuno Research, West Grove, PA), Alexa Fluor 488 donkey anti-rabbit IgG, and Alexa Fluor® 568 Donkey Anti-Goat IgG, (from Invitrogen).

### Statistical Analysis

For statistical analyses, student t-test and analysis of variance (ANOVA) were performed using GraphPad Prism, P values<0.05 were considered statistically significant. All experiments were performed independently a minimum of three times and some a maximum of five times. Each experiment contained 3 animals per group.

## SUPPLEMENTARY FIGURES AND TABLES


