# Supplementary material for: Dclk1 facilitates intestinal tumor growth via enhancing pluripotency and epithelial mesenchymal transition
Source: Oncotarget. 2014 Sep 2;5(19):9269–80. doi: 10.18632/oncotarget.2393 (PMC4253433; doi:10.18632/oncotarget.2393)
Supplement: Supplementary file 1 [file oncotarget-05-9269-s001.pdf]

SUPPLEMENTARY FIGURES AND TABLES

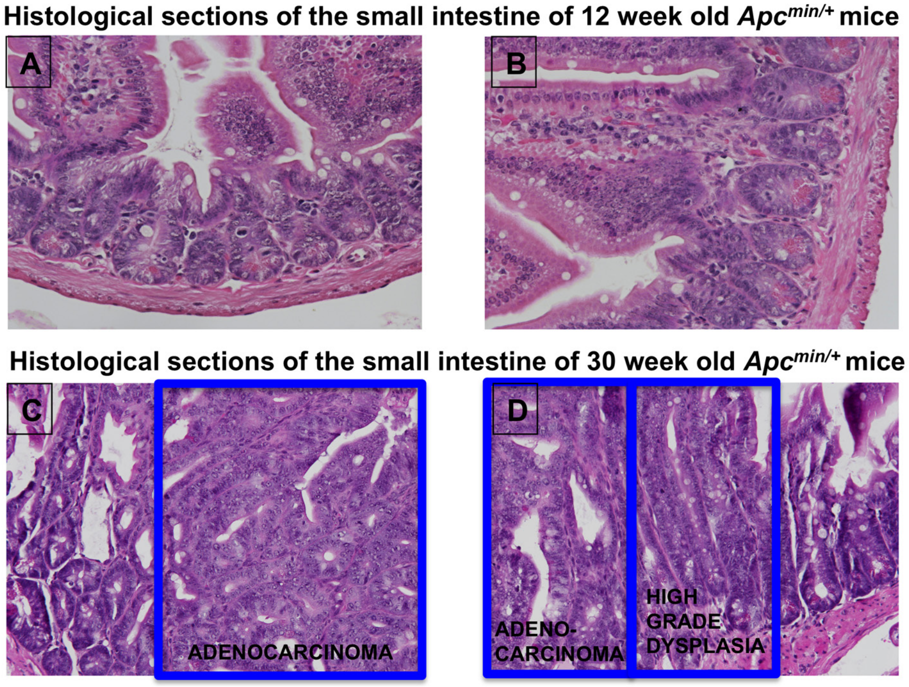

Supplementary Figure S1: Pathology of the small intestine of 12 and 30 week old *Apc*$^{Min/+}$ mice.

Apc$^{Min/+}$ mice (12 weeks old)	Apc$^{Min/+}$ mice (30 weeks old)
Animal - Active	Animal - Active
Intestinal polyps are small and sparse	Intestinal polyps are large and numerous
Crypt architecture occasionally irregular	Crypt architecture distorted
5-20% hyperplastic polyps and 0-1% low grade dysplasia and 0% adenocarcinoma	30-60% low grade dysplasia, 20-40% high grade dysplasia and 5-15% adenocarcinoma
Intestinal adenomas	Intestinal adenocarcinomas

(A & B) are the cross sections of the small intestine of 12 week old *Apc*$^{Min/+}$ mice showing hyperplastic crypts. **(C & D)** are the cross sections of the small intestine showing adenocarcinoma and high grade dysplasia of 30 week old *Apc*$^{Min/+}$ mice. C. The boxed area, occupying approximately 60% of the slide, represents an area of intramucosal moderately differentiated adenocarcinoma. D. The boxed area on the left, occupying approximately 35% of the slide, represents an area of well to moderately differentiated intramucosal adenocarcinoma. The boxed area in the center, occupying approximately 35% of the slide represents high grade dysplasia.

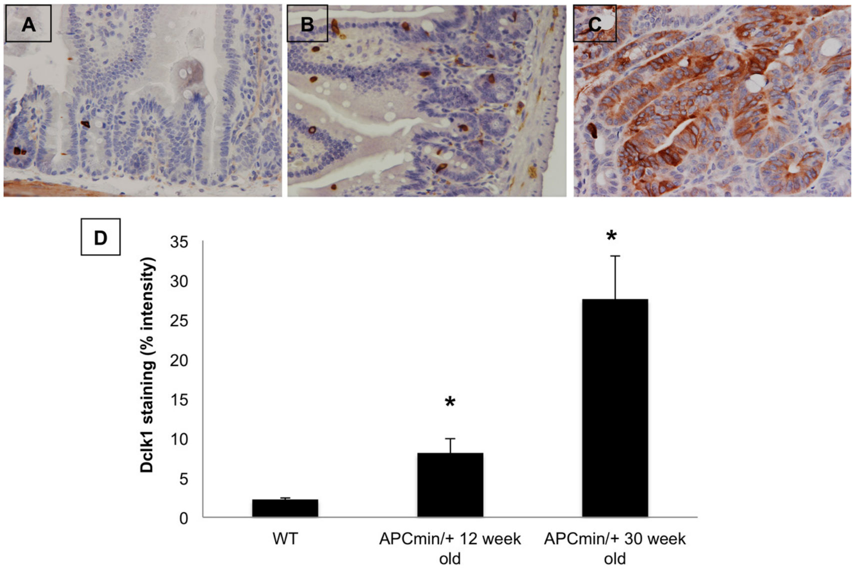

Supplementary Figure S2: **Dclk1 staining and percentage intensity between 12 week and 30 week old *Apc*$^{Min/+}$ mice and WT.** IHC for Dclk1 staining in the cross sections of small intestines of WT and 12 week and 30 week old *Apc*$^{Min/+}$ mice. **(A)** represents WT; **(B)** represents *Apc*$^{Min/+}$ mice at 12 week of age; **(C)** represents *Apc*$^{Min/+}$ mice at 30 week of age; **(D)** bar graph represents the % intensity. Immunoreactivity was evaluated by pathologists. Staining intensity was evaluated using ImageJ (NIH free software).

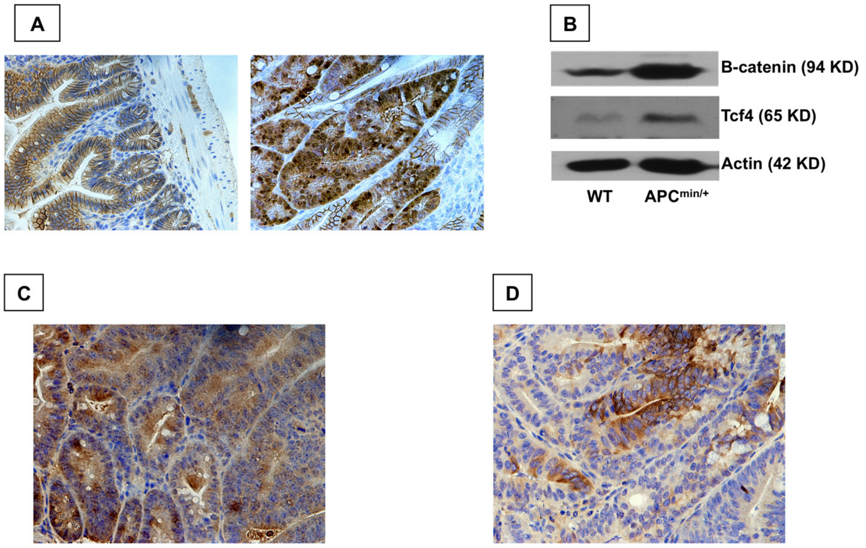

Supplementary Figure S3: IHC for β-catenin, nanog and snail in the small intestinal cross sections, and protein expression analysis for β-catenin and Tcf4 in the IECs of 30 week old *Apc*$^{Min/+}$ mice. IHC staining of β-catenin in the cross sections of small intestines of WT and 30 week old *Apc*$^{Min/+}$ mice **(A)**, β-catenin stained in the membrane of WT intestine, whereas nuclear staining was greater in the 30 week old *Apc*$^{Min/+}$ mice. Protein expression levels of β-catenin and its downstream molecule Tcf4 in the isolated IECs of WT and *Apc*$^{Min/+}$ mice **(B)**. Staining for Snail **(C)** and Nanog **(D)** in the cross sections of small intestines of WT and 30 week old *Apc*$^{Min/+}$ mice.

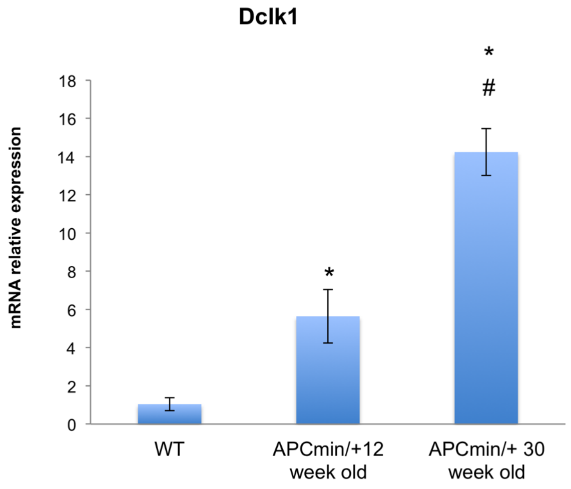

Supplementary Figure S4: Differential expression of Dclk1 in the isolated IECs of 12 and 30 week old *Apc*$^{Min/+}$ mice. Bar graph represents the mRNA level of Dclk1 in the intestinal epithelial cells (IECs) of WT and 12 week and 30 week old *Apc*$^{Min/+}$ mice. Statistically significant differences were determined by one-way ANOVA followed by Duncan's multiple comparison test. $p<0.05$ was considered statistically significant. * : compared with WT; # : compared with *Apc*$^{Min/+}$ mice 12 week old.

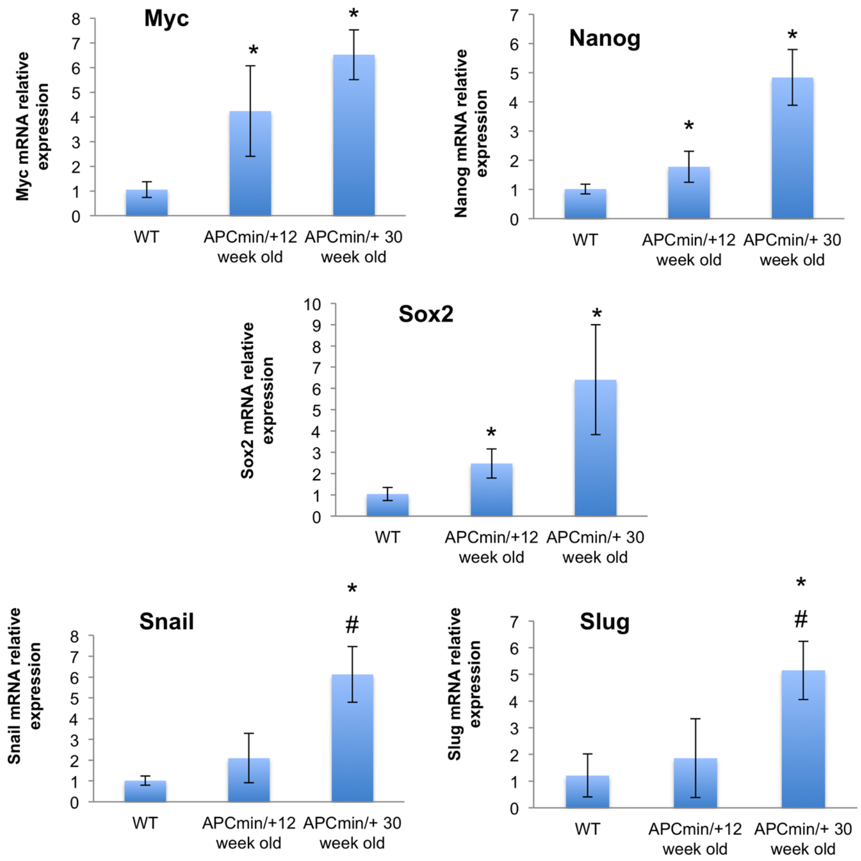

Supplementary Figure S5: Expression of pluripotency and EMT factors in the isolated IECs of 12 and 30 week old *Apc*$^{Min/+}$ mice. Bar graph represents the mRNA level of pluripotency factors Myc, Nanog and Sox2 and the EMT associated factors Snail and Slug in the intestinal epithelial cells (IECs) of WT and 12 week and 30 week old *Apc*$^{Min/+}$ mice. Statistically significant differences were determined by one-way ANOVA followed by Duncan's multiple comparison test. $p<0.05$ was considered statistically significant. * : compared with WT; # : compared with *Apc*$^{Min/+}$ mice 12 week old.

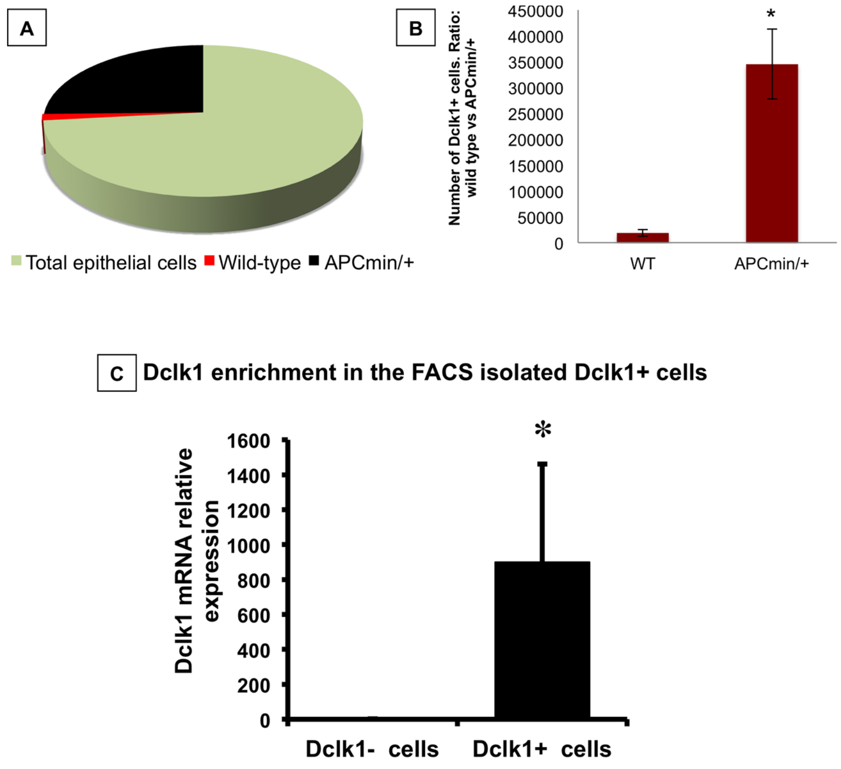

Supplementary Figure S6: Relative number of Dclk1+ cells in the small intestine of 30 week old *Apc*$^{Min/+}$ mice. **(A)** Represents the ratio of Dclk1+ cells from the small intestine between WT (2%) and *Apc*$^{Min/+}$ mice (25%) compared to the total number of IECs (100%). **(B)** Represents the number of Dclk1+ cells sorted between WT and *Apc*$^{Min/+}$ mice. **(C)** Represents the Dclk1 enrichment in the FACS isolated Dclk1+ cells of *Apc*$^{Min/+}$ mice.

Supplementary Table S1. PCR primers to miRNAs

Gene	Forward	Reverse
Let7a	5′-GAGGTAGTAGGTTGTATAGTGTTGAA-3′	5′-AAAGCTAGGAGGCTGTACA-3′
miR30a	5′-GCGCCTGTAAACATCCTCGAC-3′	5′- GCAGCTGCAAACATCCGACT-3′
miR144	5′-GCTGGGATATCATCATATACTG-3′	5′-CGGACTATTACATCATCTATACTG-3′
miR143/145	5′-GGCCTCCACCTCCGAAGA-3′	5′-GGAATCCTACCAGCCATCACTCT-3′
miR200a	5′-GCATCTTACGGACAGTGCT-3′	5′-GTCACCTTTGAACATCGTTACC-3′
miR17	5′-GTCAGAATAATGTCAAAGTGCTTAC-3′	5′- GTCAGCATAATGCTACAAGTGCC-3′
miR21	5′-GCTTATCAGACTGATGTTGACTG-3′	5′-CAGCCCATCGACTGGTG-3′
miR31	5′-CTGGAGAGGAGGCAAGATGC-3′	5′-TCAGACAGGAAAGATGGCAATAT-3′
miR98	5′-GGTAGTAAGTTGTATTGTTGTGG-3′	5′- ATGCCACACACCAAGGAAAGTA-3′
miR182	5′-ACCATTTTGGCAATGGTAGAAC-3′	5′-ACCATAGTTGGCAAGTCTAGAA-3′
U6	5′-CTCGCTTCGGCAGCACA -3′	5′-AACGCTTCACGAATTTGCGT-3′

Supplementary Table S2. Gene specific RT-PCR primers

Gene	Forward	Reverse
Dclk1	5′-CAGCAACCAGGAATGTATTGGA -3′	5′-CTCAACTCGGAATCGGAAGACT-3′
Myc	5′-AGCAACAACCGCAAGTGCT-3′	5′-GTGTCCGCCTCTTGTCGTT-3′
Nanog	5′-TCTCCTCCATTCTGAACTG -3′	5′-TGCTGGGATACTCCACTGGT-3′
Sox2	5′-GAGTGGAAACTTTTGTCCGAGA-3′	5′-GAAGCGTGTACTTATCCTTCTTCAT-3′
Snail	5′-GCCACGTCCGCACCCACACTG-3′	5′-AGGATGGGGAGGTAGCAGGG-3′
Slug	5′-GGACACATTAGAACTCACACT-3′	5′-GTAAACACTGGTTGCGCCAC-3′
Actin	5′-GGTGATCCACATCTGCTGGAA-3′	5′-ATCATTGCTCCTCCTCAGGG-3′
